# The Assimilation of Assistive Technology in Residential Care Centers for People with Intellectual Disabilities

**DOI:** 10.1100/tsw.2004.16

**Published:** 2004-03-12

**Authors:** Eli Carmeli, Carmit Cahana, Joav Merrick

**Affiliations:** ^1^ Department of Physical Therapy, Sackler Faculty of Medicine, Stanley Steyer School of Health Professions, Tel Aviv University, IL-69978 Ramat Aviv, Israel; ^2^ Office of the Medical Director, Ministry of Social Affairs, Jerusalem, Israel; ^3^ National Institute of Child Health and Human Development, Office of the Medical Director, Division for Mental Retardation, Ministry of Social Affairs, Jerusalem and Zusman Child Development Center, Divisions of Pediatrics and Community Health, Ben Gurion University, Beer-Sheva, Israel

**Keywords:** physiotherapy, assistive technology, intellectual disability, mental retardation, developmental disability, public health, Israel

## Abstract

People with intellectual disability (ID) require special support in order to achieve independence in their daily life. Persons with ID are less exposed to assistive technology, although studies have shown that the availability of aids afford an opportunity to reach independence and cooperation. The aim of this study was to examine the nature of the relationship between involvement of the physiotherapy (PT) team and the degree to which assistive technology was used. A questionnaire was sent to all PTs employed at all 54 residential care centers for persons with ID of the Division for Mental Retardation at the Ministry of Social Affairs in Israel. A significantly positive correlation was found between the degree of involvement of the PT and the utilization of assistive technology. The study results may be summarized by stating that PTs demonstrated a great deal of involvement, particularly in relation to the extent of their work in the residential care centers. PT's awareness of the importance was indicated as the major reason to use assistive technology.

## INTRODUCTION

The development of assistive technology is part of the effort made by society to cope with physical, cognitive, and sensory deficiencies causing disability, with the aim of improving and preserving quality of life[1]. Unfortunately, it is generally acknowledged, though not empirically validated, that assistive technology aids are underutilized by people with intellectual disabilities (ID)[2]. The physiotherapist (PT) has an important role in the assessment — finding the right device and instructing, guiding, and supervising the person and the care staff on the use of assistive technology. People with ID have a greater need for support than other groups of people with disabilities due to their mental, cognitive, and social limitation, in addition to any physical disabilities[3,4]. The challenge of using assistive technology is not only technical, but rather aimed at promoting improvement of quality of life[5,6].

Previous publications indicated a number of reasons why individuals with ID underutilize assistive technology and explained the existing barriers to this outcome[7,8]. On the other hand, we do not know of any study published on the role of the PT in addressing assistive technology to people with ID. The PT, the direct support staff member, and the user must know about the advantages and the limitations of the use of aids. For this purpose, it is important to evaluate the disability of the user and the support required of the technological aids. It is imperative for the PT to perform a comprehensive functional diagnosis, proper instruction, encouragement, supervision, and support, on receiving the instrument. A person with ID requires much attention and a longer period of practice and acclimation in order to know how to use the new instrument.

The aim of this study was to examine the nature of the relationship between involvement of the PT team and the degree to which assistive technology was used in residential care centers for persons with ID in Israel. Investigating this question may help the PT team to develop strategies of evaluation, instruction, supervision, and operation of treatment programs. The target would also be to use assistive technology aids, with the cooperation and direct support of the staff, to maximize implementation and the use of these aids in a daily schedule of people with ID.

## METHODS

A cross-sectional descriptive study was conducted in all the 54 residential care centers in Israel, with a total population of 6,000 people, under the approval of IRB of Tel Aviv University, the National Institute of Child Health and Human Development, Office of the Medical Director and Division for Mental Retardation, Israel.

We reviewed various questionnaires to identify themes that we believed should be part of an assistive technology survey for disabled people. From this review, a questionnaire containing 38 closed items in two parts was developed. The first part had personal, demographic, and professional questions, while the second part presented questions with five possible response categories (very regularly, regularly, occasionally, infrequently, never) in order to get information regarding the extent of the involvement of PT team and the degree to which assistive technology was used. The questionnaire was designed to be administrated by a PT and its context addressed questions regarding type of aids, way of usages and degree of satisfaction, existence of diagnosis process, evaluation, and guidance (i.e., documentation, reports, follow-up, maintenance, and repair). Inter-rater reliability between four experts was high (ICC = 0.91; r = 0.90). All four experts agreed that the content domains (i.e., assistive technology used) had been addressed adequately, thus content validity was supported. Percentage agreements (Kappa statistic for test-retest reliability) for categorical variables ranged between 82 and 94%.

### Data Analysis

Data analysis was performed using SPSS version 10. Frequencies, means, and standard deviations were used to describe the data. Multiple regression analyses were conducted to explore variables that might predict patterns of PT involvement in which assistive technology was used. To analyze the relationship between single variables, we used $X^2$ tests. All tests were performed with a type I error rate of 5%.

## RESULTS

Of 92 questionnaires mailed out to all PTs, 70 were returned, however, 5 of those returned were partially blank. Therefore, we received 65 usable questionnaires, a 71% response rate based on the initial mailing. The degree of involvement in the workplace is a function of the extent of the PT position in each setting. Among the 65 PT respondents who provided personal and professional information, 75% were women, 75% held BA degree, and 69% worked part time for at least 3 years in the residential care center (Table 1).

**TABLE 1**
**Sociodemographic Data of the PT (n = 65) and Residential Care Center (n = 54)**

| Variable | Outcomes | No. | % | $X^2$ | *p* |
|---|---|---|---|---|---|
| Gender | Male | 16 | 25 | 3.27 | 0.005 |
| | Female | 49 | 75 | | |
| Education | LPT | 13 | 20 | 54.84 | 0.05 |
| | BA in PT | 35 | 75 | | |
| | MSc in PT | 3 | 5 | | |
| Working time (h/week) | Up to 10 | 29 | 42 | 25.63 | NS |
| | 11–20 | 32 | 45 | | |
| | 11 > | 4 | 8 | | |
| Working experience (months) | Up to 12 | 22 | 30 | 33.28 | |
| | 12–36 | 35 | 58 | | 0.05 |
| | 37 > | 7 | 11 | | |
| Residential care center | Government | 14 | 26 | | |
| | Public | 17 | 31 | | |
| | Private | 23 | 43 | 4.1 | 0.05 |

In Israel, the Division for Mental Retardation (DMR) provides residential care service for all persons with ID through government, public, and private centers. The clients, the budget, and the supervision are provided by the government (DMR), but the ownership of the center can be either government, public (not-for-profit), or private. Table 2 demonstrates the distribution of assistive technology in privately owned and government-owned residential care centers. Most devices were significantly used ($p < 0.05$) in privately owned residential care centers. The majority of the participants (i.e., permanent residents) used either splints or corsets along with one of the four walking aids.

From Table 3, it can be seen that most of the PTs (45%) used the assistive technology for therapeutic gait, while only 5% used this technology for sensory stimulation.

Tables 4 and 5 demonstrate the involvement of the PT and the mechanism used to promote assistive technology within the 54 residential care centers. The role of PT in residential care centers in assimilating rehabilitative technology is crucial. It includes screening (58%), analysis of findings, recording of documentation (50%), ordering the device, monitoring and supervising the on-going use (65%), and staff instructions, individually based (50%). Continued quality assurance (CQA) was the mechanism used for monitoring the implementation of assistive technology (Table 4). A good correlation was found between use of the equipment and involvement of the PT (r = 0.50, $p < 0.001$), as well as good correlation found between the degrees of recorded use of aids and the degrees to which aids were used in the residential care centers (r = 0.63, $p < 0.001$).

**TABLE 2**
**The Distribution Ratio between Assistive Technology in Private vs. Government-Owned Residential Care Centers in Israel (n = 6,000)**

| Variable | Total used (%) | Government (%) | Private (%) |
|---|---|---|---|
| Splint or corset | 60 | 0 | 100 |
| St. Walker | 48 | 80 | 20 |
| Rollator | 52 | 20 | 80 |
| Arms rest walker | 38 | 100 | 0 |
| Quad cane | 17 | 50 | 50 |
| Limb mobilizer | 27 | 0 | 100 |
| Other | 32 | 20 | 80 |
| *p* | | | 0.05 |

**TABLE 3**
**Distribution of Assistive Technology Main Used Purposes**

| Variable | Purposes (%) |
|---|---|
| Functional gait | 20 |
| Therapeutic gait | 45 |
| Preventive | 20 |
| Sensory stimulation | 5 |
| General activity | 10 |

**TABLE 4**
**The Present Mechanism of Assistive Technology**

| Variable | Outcome | % | $X^2$ | *p* |
|---|---|---|---|---|
| Existing records | Updated/outdated | 50/50 | 0.429 | NS |
| Initial screening | Up to 50% | 35 | | |
| | 51% > | 65 | 12.71 | NS |
| Done by | PT | 58 | | |
| | Non-PT | 42 | 6.08 | NS |
| Inservice* | Workshop | 25 | | |
| | Single meeting | 20 | 4.10 | |
| | Individual | 50 | | 0.05 |
| | Group | 35 | | |
| On-going maintenance responsibility* | Administrator | 28 | | |
| | Caregiver | 32 | 0.027 | NS |
| | Aide | 25 | | |
| | PT | 55 | | |
| On-going follow-up responsibility | PT | 62 | | 0.05 |
| | Non-PT | 48 | 33.11 | |
| Method of follow-up and supervision* | CQA | 65 | 0.78 | 0.05 |
| | Randomized | 35 | | |
| | Interview | 30 | | |

* The PT could mark two answers.

**TABLE 5**
**Characteristics of Assistive Technology Used**

| Variable | | Outcome (%) | $X^2$ | *p* |
|---|---|---|---|---|
| Actual time-used by the resident | Up to 25% | 20 | 2.78 | |
| | 26–50% | 30 | | |
| | 51–100% | 50 | | 0.05 |
| Reason **not** to use* | Environmental | 38 | 19.70 | NS |
| | Treatment time | 25 | | |
| | Product deficiency | 20 | | |
| | Other | 17 | | |
| | Very regularly | 18 | 11.22 | NS |
| Product satisfaction | Regularly | 31 | | |
| | Occasionally | 16 | | |
| | Infrequently | 22 | | |
| | Never | 13 | | |

* The PT could mark two answers.

Table 5 demonstrates the percentages used or not used of the assistive device and how PTs were satisfied with the products. It seems that most residents actually used their aid, and the major reasons not to use them were environmental barriers (38%) and short treatment time (25%). The PT graded their overall satisfaction from the function and efficiency of the assistive aid as “regularly” (31%), i.e., it answers their expectations, and 13% answered that the assistive aid never satisfied them. A good correlation was found between the extent to which the use of aids was recorded and the involvement of the PT ($r = 0.70$; $p < 0.001$).

## DISCUSSION

The total results of this study indicated a large degree of involvement by PTs in assistive technology despite their limited number of work hours per week. This fact apparently explains the significance of the technology as seen by the PT team and the direct support staff in the residential setting in Israel. We believe that the significance of the issue of assistive technology as seen by PTs probably stems from their academic background and professional training, but not necessarily from their years of experience. The variety of technology aids in residential settings for people with ID, which included aids that were technologically simple and/or complex, was interesting because a literature review found that residents with ID usually were not exposed sufficiently to complex assistive technology equipment and therefore the use of these aids in this population is limited and minimal[3,5]. In contrast, our study found that most of the residents in residential settings in Israel used a variety of assistive aids, a fact indicating high awareness among the direct support staff on the one hand, and the resident's responsiveness to the personal accessory adapted to him/her, on the other hand.

According to our results, evidence showed that the maximal use of supportive equipment was aimed at purposes of mobility and gait. The majority of PTs reported high prevalence of therapeutic walking, indicating both individual therapy and the choice of a “work tool”, which is an assistive technological aid. In addition, it was found that the use of assistive technology had additional purposes not reported in the literature, such as preservation of existing status and treatment of involuntary movements.

Deficient documentation of findings and of the constant supervision was evident from the study results. The PTs were not strict about updating and recording their findings in the resident's file. We believe that the supervision and operation of a structured therapy program, through rehabilitative assistive technology, should usually be performed by staff from the health services professions, but we found that the supervision was often performed by people without a medical background, such as educational counselor or even a representative of the management of the residential setting. This phenomenon had implications for the monitoring stage of the daily use of aids. Thus the responsibility for continuous operation of rehabilitative technology falls almost equally on the management on the one hand and the PT team on the other. This division, however, did not have a negative effect on the use of rehabilitative aids and the degree of use among most of the residents.

An ethical aspect was raised from the study outcomes. The importance of paperwork has been discussed extensively and recommended by the American Physical Therapy Association[9]. Documentation of the findings is indispensable in order to record milestones in the patient's condition and to facilitate interdisciplinary communication. Moreover, a precise recording is a basic standard for provision of efficient and beneficial comprehensive rehabilitation. Deficient, outdated, and imprecise recording is harmful to the quality of care. We believe that the introduction of a uniform system of recording for all stages of the PT involvement in the assimilation of rehabilitative technology may contribute to more efficient and beneficial use of the various aids.

The extent to which the residents used assistive technology aid was wide and varied. Our findings showed that the PT opinion for lack of use or barrier to utilization of assistive technology had multifactorial reasons, such as insufficient support, inability to reach individual adaptation, and maintenance difficulties. Most PTs indicated that residents did not realize their maximal functional ability due to minimal use of aids. The reasons for minimal use of aids were varied and included, to a similar degree, environmental conditions that make it difficult to use them and organization of a therapy system (i.e., the time allocated to a therapy and practice unit). Topography of the residential setting and allocation of accurate therapy time should expand the use of aids and thus improve the functional potential of the residents.

Despite the technological development of instrumentation, there is still place for technical improvement of the specifications, such as the weight of the item, its stability, or replacement of spare parts. Operating difficulties may cause lack of will and inability to use the equipment appropriately.

## CONCLUSIONS

In light of the findings concerning the use of assistive technology aids in residential setting for people with ID in Israel, we recommend establishing a nationwide system with mechanisms for the control and evaluation of the professional work of PTs, also in the context of assimilating assistive technology. Introducing a structured system will help the process of assimilating assistive technology in residential care centers, increase its use, and deepen the connection between the administrative staff and people in the clinical field, which may lower the direct and indirect financial costs related to the use of assistive equipment[10]. Various institutions should take part of this system, including university affiliated facilities[11].

## ACKNOWLEDGMENTS

We would like to thank Anne and Eli Shapira, Charitable Foundation, Portland, Oregon for supporting this work.

## BIOSKETCHES

**Eli Carmeli, BPT, PhD**, is currently a Senior Lecturer of Gerontology and Anatomy in the Department of Physical Therapy Department, Stanley Steyer School of Health Professions, Sackler Faculty of Medicne, Tel Aviv University. He is also the Editor-in-Chief of the Israeli Journal of Physiotherapy Society. Eli received his BPT degree from the Sackler Faculty of Medicine, Tel Aviv University and his PhD degree from the Rappaport Faculty of Medicine at the Technion in Haifa. Postdoctoral research was conducted at the University of Florida in Gainesville. He is investigating the aging process both on the cellular and clinical level. E-mail: elie@post.tau.ac.il Website: http://www2.tau.ac.il/Person/medicine/HealthSchool/researcher.asp?id=agfhfiffl

**Carmit Cahana, BPT, MSc,** is the chief physiotherapist at the Office of the Medical Director, Division for Mental Retardation, Ministry of Social Affairs, Jerusalem, Israel. She is interested in research about the role of the physiotherapist and persons with intellectual disability, assistive technology, Snoezelen or sensory multistimulation, and service utilization. E-mail: carmit_c@netvision.net.il

**Joav Merrick, MD, DMSc,** is Professor of Child Health and Human Development affiliated with the Zusman Child Development Center, Division of Pediatrics and Community Health at the Ben Gurion University, Beer-Sheva, Israel; the Medical Director of the Division for Mental Retardation, Ministry of Social Affairs, Jerusalem; and the Founder and Director of the National Institute of Child Health and Human Development. He has numerous publications in the field of child and human development, rehabilitation, intellectual disability, disability, health, welfare, abuse, advocacy, quality of life, and prevention. Dr. Merrick received the Peter Sabroe Child Award for outstanding work on behalf of Danish Children in 1985 and the International LEGO-Prize ("The Children's Nobel Prize") for an extraordinary contribution towards improvement in child welfare and well being in 1987. E-mail: jmerrick@internet-zahav.net. Website: www.nichd-israel.com
